# Two Major 21^st^ Century Public Health Challenges

**DOI:** 10.3934/publichealth.2016.3.573

**Published:** 2016-08-15

**Authors:** Christopher Alan Birt

**Affiliations:** Department of Public Health and Policy, University of Liverpool, UK

Inequalities in health pose the challenge supreme over all others to public health professionals in developed countries. The “Black Report”, the product of the committee set up by the UK Government in the late 1970s to investigate health inequalities in UK, was published in 1980 [1]. It opened the eyes of a generation to the extent of the health gap between the health experienced by the more prosperous in developed countries and that of the more deprived parts of such populations. This is demonstrated by very marked differences in life expectancy of those living in prosperous parts of our cities as compared with that of those living in more deprived areas. In London it has been shown that life expectancy falls steadily, tube station by tube station, as one travels eastward on the District Line from the West End; similar findings have been demonstrated in many other cities; Molony and Duncan [2] have described analogous findings in Glasgow, where a traveller on the suburban train line service between Jordanhill, in the West End, and Bridgeton in the East End, would pass through a two year reduction in life expectancy between each adjacent stations where the trains stop along the line.

Since the “Black Report” there have been many similar studies in various European countries, including the UK [1],[3],[4]. Actually, Syme and Berkman [5] had published similar findings in the USA as early as the 1970s, but it seems that these reports were considered so shocking at that time, and outside the bounds of appropriate scientific enquiry, that they were almost hidden away, and treated almost as “samizdat literature” (as described by Marmot [6]). However, Marmot himself and his colleagues have thrown considerable light on health inequalities through their reports on the social determinants of health [7]–[9], which include the main aetiological factors responsible for health inequalities. Meanwhile, Pick and Wilkinson [10] have shown us that, in countries where the gap between rich and poor is narrow (such as in Sweden or Japan), the health status of everyone (including the rich) is superior to that of everyone in countries where the gap between rich and poor is much wider (such as in USA and UK). On the other side of the Atlantic, Deaton has written a very readable account of many of the issues concerning health, wealth and inequality [11], including a useful historical overview of the subject.

Marmot [6] has provided many potential entry points at which public health workers might obtain entry into these problems, bringing public health skills and approaches to bear on at least limited aspects of them. Molony and Duncan have described the health inequalities situation in Scotland, and how this is being addressed there. However, such activity in reality can provide little more than tinkering around the edges of the matter; inequalities of health and the social determinants responsible for these are the outcome of the economic system prevalent in the developed world, and ultimately the solutions can only really be economic ones. Such evidence as there is indicates that health inequalities were much narrower in all western countries when Keynesian economics reigned supreme, from 1945 to 1975, and then they began to widen, and have continued to do so, as neoliberal economic policies replaced Keynesian ones [12]. There are some signs, both in north America and in Europe, that neoliberal policies are being questioned ever more severely; maybe we are entering an era when economics ministers may prove to be more responsive than in recent history to the health needs of the more deprived parts of the populations of developed countries.

The other major challenge to public health consists of nutrition, and the major policy areas now inevitably associated with it. Our most major health problems are caused either by over-nutrition and obesity or by malnutrition [13],[14]. Hogler and colleagues [13] remind us of the need to continue to address malnutrition, including in developed countries, while Xiaohui Hou demonstrates the importance of addressing, in particular, maternal and child under-nutrition in developing countries [14]. Birt [15],[16] has described the extent to which in Europe there is almost a mismatch between the food grown and produced (agricultural policy) and the types of food most needed by European populations for their healthy nutrition, and Pushkarev [17] has described how the EU, through reform of the Common Agricultural Policy, should build public health nutrition into this. Meanwhile, over the last 30 years there has been an increasing awareness of the environmental threats posed by modern farming practices. These are numerous, but have become especially visible now we are aware that farming contributes more global warming gases to the atmosphere than does any other industry [18], with dairy and beef production being the cause of most of this. It is therefore interesting to observe that, while a nutrition-friendly food policy in both Europe, North America, and Australia, etc., would necessitate a reduction in beef and dairy production and consumption, with increased production and consumption of fruit and vegetables, such policy movement would also be consistent with environmental protection and reduction in global warming gas production [19]. Accordingly, it is at last becoming recognised that we need to develop policies for sustainable healthy nutrition, to incorporate together agricultural and food industry policy, public health nutrition policy, and policy for environmental sustainability [20]. O'Flaherty and Guzman [21] have described how there are lessons to be learned from other public health successes, such as in the case of tobacco; they also describe how much there is to do to encourage the food industry to comply with objectives to provide our populations with much healthier food products than are many of those they sell currently, especially in the context of any meaningful attempts to address seriously the world`s obesity epidemic.

Health inequalities and the social determinants of health, and sustainable healthy nutrition, both provide major challenges to the manner in which developed societies and countries are organised. It remains to be seen whether the advocates promoting both sustainable healthy nutrition and policies designed to address the social determinants of health can obtain sufficient prominence amongst all political priorities so as to effect the genuine changes in public policy which are needed, if these public health challenges are at last to be met effectively. If this is not achieved, this can only indicate the relative ineffectiveness of public health in the twenty first century.
